# Supplementary material for: Employer Actions in Office Settings and Women’s Perception of the Workplace as Supportive of Healthy Eating: A Cross-Sectional Pilot Study
Source: Nutrients. 2024 Nov 1;16(21):3766. doi: 10.3390/nu16213766 (PMC11547438; doi:10.3390/nu16213766)
Supplement: Supplementary file 1 [file nutrients-16-03766-s001.zip › nutrients-3277205-supplementary.pdf]

Supplementary data

Workplace Healthy Eating Scale (WHES), developed by A. Hyży

Please read the following statements and then indicate the extent to which you agree with them using a 5-point Likert scale (1 = strongly disagree, 5 = strongly agree).

Remember that there are no right or wrong answers. Please be honest.

	**Items**	**1**	**2**	**3**	**4**	**5**
**1**	My employer takes care to ensure that I eat well					
**2**	My employer provides me with access to healthy food at the workplace					
**3**	The activities undertaken by my employer regarding healthy nutrition of employees are sufficient					
**4**	My employer takes care of addiction prevention at the workplace					
**5**	At my workplace, activities related to healthy eating (lectures, workshops, consultations) take place frequently enough					
**6**	The atmosphere at my workplace is conducive to a healthy lifestyle and healthy eating					
**7**	My direct supervisor supports me in making the right nutritional choices					
**8**	The management at my workplace supports me in making the right nutritional choices					
**9**	Employee health is my employer's priority					
**10**	My co-workers support me in making the right nutritional choices					
